# Unraveling the Individualized Shared and Distinct Dynamic Functional Connectivity in Idiopathic Autism Spectrum Disorder and Fragile X Syndrome

**DOI:** 10.1002/cns.71046

**Published:** 2026-07-24

**Authors:** Boli Pan, Junbo Wang, Danyong Feng, Yiting Zhu, Ning Tang, Dongyun Li, Qiong Xu, Rihui Li

**Affiliations:** ^1^ Centre for Cognitive and Brain Sciences, Institute of Collaborative Innovation University of Macau Macau SAR China; ^2^ Department of Radiology Children's Hospital of Fudan University Shanghai China; ^3^ Department of Electrical and Computer Engineering, Faculty of Sciences and Technology University of Macau Macau SAR China; ^4^ Department of Child Health Care Children's Hospital of Fudan University Shanghai China

**Keywords:** ASD, dynamic functional connectivity, FXS, normative modeling, personalized evaluation

## Abstract

**Aims:**

Autism Spectrum Disorder (ASD), a neurodevelopmental disorder, and Fragile X Syndrome (FXS), an X chromosome‐linked hereditary neurodevelopmental disorder, exhibit overlapping phenotypes like social impairment and stereotyped behaviors. Previous studies have predominantly explored the functional network characteristics of either FXS or ASD. However, the shared and distinct dynamic neural circuitry underlying both disorders remains unclear, impeding personalized treatment.

**Methods:**

This study examined the shared and distinct temporal topological properties of ASD and FXS, along with their respective patterns of functional network deviation relative to typically developing (TD). A total of 47 FXS children (5.2 ± 2.5 years), 91 ASD children (3.37 ± 1.37 years), and 52 TD children (5.2 ± 2.9 years) were included.

**Results:**

Analyses revealed both shared hyperconnectivity involving sensorimotor network (SMN), default mode network (DMN), ventral attention‐visual network (VAN‐VN) connectivity, and subcortical‐cerebellum network (SN‐CN) and distinct temporal topological features, with temporal metrics differences primarily localized to intermediate activation patterns linked to cognitive switching.

**Conclusions:**

As a large‐scale pediatric dFC study comparing FXS and ASD, it advances mechanistic understanding and proposes novel differentiation.

## Graphical abstract

Enhancing the prediction accuracy of clinical symptoms in ASD and FXS through analysis of Shared and Distinct Individualized Dynamic Functional Connectivity Patterns. (A) Dynamic functional connectivity (dFC) pattern identification. (B) Modeling based on dFC patterns and demographic features. (C) Accurate prediction of clinical symptoms by normative models.

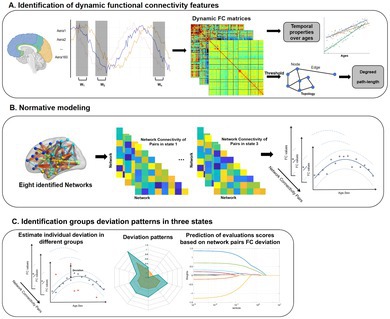

## 1 Introduction

Autism Spectrum Disorder (ASD) is a neurodevelopmental disorder caused by a variety of risk factors [1], whereas Fragile X Syndrome (FXS) is an X chromosome‐linked genetic disorder [2]. Both disorders exhibit overlapping phenotypes such as social impairments and sensory abnormalities [3], making them difficult to distinguish through clinical assessment alone [4]. Despite these shared phenotypes, there are critical differences between the two disorders. FXS is caused by a well‐characterized trinucleotide repeat expansion in the Fragile X Messenger Ribonucleoprotein 1 (*FMR1*) gene, resulting in transcriptional silencing and subsequent loss of fragile X messenger ribonucleoprotein [5, 6]. In contrast, ASD encompasses a wide range of neurodevelopmental variations with complex etiologies involving intricate gene–environment interactions and epigenetic modifications [1, 7, 8]. This pathophysiological distinction underscores the importance of elucidating both convergent and divergent neural signatures in ASD and FXS. Specifically, by studying FXS, we can gain insights into specific neural mechanisms contributing to cognitive‐behavioral impairments, offering a focused perspective to understand broader ASD heterogeneity.

Recent neuroimaging investigations have shown that brain function emerges not from isolated regions but through dynamic interactions within and between intrinsic functional networks [9, 10]. In particular, resting‐state functional connectivity (rsFC) has been widely used to characterize altered functional connectivity (FC) at the whole‐brain level, or in intrinsic networks such as the default‐mode network (DMN) across various populations [11, 12, 13], such as ASD and FXS [14, 15, 16]. For example, previous rsFC studies have reported enhanced FC of DMN in ASD individuals, which was associated with social communication deficits and repetitive behaviors [14, 17]. Hyperconnectivity of the sensorimotor network (SMN) has also been linked to the aberrant sensory processing in ASD [18, 19]. Graph theory‐based topological analyses also revealed increased modularity and reduced global efficiency in the brain networks of the ASD population [20, 21]. On the other hand, emerging evidence showed that FXS predominantly affected the subcortical network (SN) and DMN [15, 22], with structural involvement spanning the prefrontal cortex, superior/middle temporal gyri, caudate nucleus, hippocampus, and amygdala [2, 3, 23, 24].

Traditional rsFC analysis relies on the assumption that functional connectivity among different brain regions remain static over time [25, 26], yet accumulating evidence suggests dynamic functional connectivity (dFC) fluctuations at millisecond timescales that better capture neurocognitive processes [25, 26]. This approach shows promise for advancing our understanding of resting‐state brain activity and may offer important insights into a range of brain conditions [16, 27, 28]. In particular, dFC analysis has demonstrated good reliability in characterizing the brain's dynamic temporal patterns [29] and capturing cognitive switching processes that are highly relevant to psychiatric research [30]. Given the divergence in developmental trajectories of ASD and FXS compared to the typically developing (TD) individuals [3, 31, 32, 33], combining dFC measures with computational modeling could quantify individual deviations from TD patterns while controlling for covariates like age and sex [34, 35, 36]. Such an approach may establish the relationship between the time‐varying brain network and clinical manifestations [36], supporting personalized evaluation and prediction for affected individuals.

In this study, we aimed to investigate the common and shared dFC patterns in idiopathic ASD and FXS children relative to TD children and to further establish a personalized model using a normative modeling approach. Regarding the overlapping phenotypes but distinct etiologies of ASD and FXS, we hypothesized that both ASD and FXS may share common and distinct dFC patterns and topological properties. We further hypothesized that dynamic network abnormalities are specifically linked to individual cognitive‐behavioral function, serving as a potential diagnostic and monitoring tool for personalized treatment.

## 2 Materials and Methods

### 2.1 Participants

This study consecutively enrolled 190 participants. The cohort comprised 47 FXS children (5.2 ± 2.5 years) confirmed through genetic testing (i.e., revealing more than 200 CGG repeats in the *FMR1* gene). Ninety‐one ASD children (3.37 ± 1.37 years) without identified genetic etiologies were included as the idiopathic ASD group, with diagnoses established via the Diagnostic and Statistical Manual of Mental Disorders, Fifth Edition (DSM‐5) and the Autism Diagnostic Observation Schedule, Second Edition (ADOS‐2). Fifty‐two TD children (5.2 ± 2.9 years) served as the comparison group. Children with cerebral palsy, or other degenerative diseases, or with recognizable lesions or abnormalities on scans confirmed by MRI, were excluded.

### 2.2 Cognitive‐Behavior Assessments

We conducted a series of physical and neurological examinations, comorbidity evaluations, and standardized diagnostic and developmental assessments, including the ADOS‐2 [37] and the Griffiths developmental scales‐Chinese version (GDS‐C) [38]. The ADOS‐2 is a semi‐structured observational assessment for ASD. It provides a comprehensive scoring system encompassing Social Affect (SA), Restricted and Repetitive Behaviors (RRB), an overall total score, and the Calibrated Severity Score (CSS), which allows for comparison across modules and individuals [37]. The GDS‐C provides a multidimensional neurodevelopmental evaluation in children aged 0–8 years, assessing gross motor (GM), fine motor (FM), language, personal‐social (PS), and performance domains to identify developmental variations or delays [38].

### 2.3 Data Acquisition and Preprocessing

All neuroimaging data were acquired using a 3.0 T GE Discovery MR750 scanner (GE Healthcare, Milwaukee, WI) with an 8‐channel phased‐array head coil. Children and parents were accommodated in a dedicated preparation room adjacent to the MRI suite with low‐intensity lighting during pre‐scan adaptation. Scans were taken 15–20 min after falling asleep to make sure the children were in a deep sleep. For children who were unable to fall asleep twice due to issues like paroxysmal dizziness or headache, a low dose of chloral hydrate (50 mg/kg) was administered with parental consent and under the supervision of licensed clinicians or anesthesiologists. T1‐weighted structural brain images were obtained sagittally using a 3D fast spoiled gradient echo (FSPGR) sequence with the following parameters: repetition time (TR) of 8.2 ms, echo time (TE) of 3.2 ms, flip angle of 12°, voxel size of 1 × 1 × 1 mm^3^, field of view (FOV) of 256 mm, matrix size of 256 × 256, and a scan duration of 510 s. Resting‐state functional images were captured using an echo planar imaging (EPI) sequence with a TR of 2000 ms, TE of 30 ms, flip angle of 90°, FOV of 22.4 cm, resolution of 64 × 64 matrix, slice thickness of 3 mm, 39 slices, no spacing between slices, bandwidth of 250 kHz, and a scan duration of 400 s.

Preprocessing of images and regions of interest (ROIs) was performed using the Data Processing & Analysis for Brain Imaging (DPABI) [39] and a customized script. We identified eight intrinsic functional networks using a well‐established functional parcellation derived from a sample of 1000 healthy individuals [40]. These networks comprise 160 ROIs, including the SMN, Ventral attention network (VAN), Visual network (VN), Dorsal attention network (DAN), DMN, frontoparietal network (FPN), SN, and cerebellum network (CN) [40]. Information about the ROIs in each network was summarized in the Table S1.

### 2.4 Dynamic Functional Connectivity Analysis

#### 2.4.1 Sliding Window Segmentation

The sliding window approach is a classical method for dFC analysis [25, 26]. We employed a tapered window of 44 s with a step size of 1 TR (2 s) and Gaussian smoothing (*σ* = 3 TRs), segmenting all participants' resting‐state signals extracted from ROIs into 169 windows. This window and step size configuration has been validated to achieve an optimal balance between matrix quality estimation and exploration of brain dynamics [26, 27, 28]. Previous studies have demonstrated that cognitive states can be effectively identified with window sizes ranging from 30 to 60 s [26, 27, 28, 41]. The tapered window design reduces boundary effects and enhances the signal‐to‐noise ratio [26, 41]. Finally, dFC matrices were derived by calculating Pearson correlation coefficients and Fisher Z‐transformed.

#### 2.4.2 Clustering Analysis

To identify recurrent FC states, we applied k‐means clustering with Manhattan distance (L1 norm), which has been validated as an effective metric for high‐dimensional data [27]. The optimal cluster number (*k* = 3) was determined via the elbow criterion [27, 28, 41]. Initially, the TD group was clustered to derive group‐level templates representing canonical neurodevelopmental states, which reflect co‐activated brain patterns at the normal population level. The resulting templates were used as fixed centroids to classify ASD and FXS groups by calculating the minimal Manhattan distance between each window and the TD‐derived centroids. We then averaged the windows of the ASD and FXS groups and finally obtained the dFC matrices under three states at the group level. This cross‐group clustering strategy eliminates inter‐group centroid variability, ensuring comparability across diagnostic categories [42].

#### 2.4.3 Temporal and Topological Properties of Dynamic Functional Connectivity

We investigated the temporal metrics of dFC by calculating three metrics: dwell time (state duration), transition frequency (number of shifts between states), and lifetimes (persistence of state sequences), and assessed group differences by incorporating group and age into regression models, and evaluated the *p*‐values of group‐effect, with false discovery rate (FDR) correction applied to control for multiple comparisons. Because childhood is a period of rapid brain development [43], we assessed associations among temporal metrics and developmental effects by using regression coefficients derived from relationships between age and each temporal metric.

For the identified dFC networks, we calculated degree and path length, as previous studies have reported significant alterations in these topological properties in individuals with ASD ([20, 21, 44]; see Supporting Information). The dFC matrices were sparsified using thresholds ranging from 0.1 to 0.4 (increment = 0.02) [45], and the area under the curve (AUC) for each graph‐theoretical metric was calculated. Group differences in these topological metrics and their AUC were then assessed by incorporating group and age into a linear model and evaluating the *p*‐values of the group effect.

### 2.5 Normative Modeling of the dFC Network

We employed a normative modeling approach to quantify individual deviation in brain‐derived dFC features relative to the TD group, from which a personalized framework can be established for the ASD and FXS groups. We parcellated the cerebral cortex into eight networks as mentioned above and computed the within‐network FC and between‐network FC for each participant. For the network FC, we estimated normative models using Gaussian process regression (GPR) to predict FC as a function of age, sex, and brain state (State 1/2/3). The GPR is a Bayesian nonparametric interpolation method, providing both point estimates and coherent measures of predictive uncertainty [46]. To evaluate model generalizability, we performed 10‐fold cross‐validation on the trained models. Finally, the normative models trained on the TD group were applied to subsequent disorder‐specific deviation analyses. In this study, 36 normative models were constructed to predict dFC patterns for each of the 36 network‐pair connectivity features, covering all possible network‐to‐network connections.

### 2.6 Individual Evaluation of Deviation Patterns in Normative Models

Using normative modeling, we obtained deviation FC values for different network pairs in three states for ASD and FXS children. We identified network pairs with significant deviation (*p* < 0.05) in different states, referred to as deviated network pairs, and calculated their Cohen's *d*. Cohen's *d* quantifies the magnitude of deviation in FC between the patient group and normative model predictions. Next, we calculated the Z scores for each participant in the ASD and FXS groups for the deviated network pairs as follows:

$$Z_{i,k}=\frac{{\mathrm{FC}}_{\text{observed},i,k}-{\mathrm{FC}}_{\text{predicted},i,k}}{\sigma_{k}}$$

where *i* and *k* denote different networks individually, and *σ*_*k*_ is the prediction uncertainty. Based on the deviation FC of the intrinsic networks, an elastic net regression model was employed to predict autistic symptoms (i.e., ADOS‐2 scores) in ASD children. Elastic net is a linear regression method that combines Ridge Regression and Lasso Regression and is particularly suitable for situations with a large number of features, multicollinearity, or the need for feature selection [47]. Fivefold cross‐validation with optimal hyperparameters was used for prediction. Finally, we calculated the correlation coefficient between predicted and true scores to assess prediction accuracy [48, 49] and computed the mean absolute error (MAE) to evaluate overall prediction performance.

### 2.7 Classification Based on Z Scores of Deviations

We applied Elastic Net regularized logistic regression to classify the ASD, FXS, and TD participants. Each subject's data from three states and 36 connections was reshaped into a 108‐dimensional feature vector, and a stratified five‐fold cross‐validation procedure was used for evaluation. Within each fold, three logistic regression models were trained using a One versus Rest strategy with an Elastic Net mixing parameter of *α* = 0.5. The optimal regularization strength was selected via internal cross‐validation. Class probabilities were derived using the logistic function, and the final label was assigned based on the highest predicted probability. Model performance was quantified using accuracy and One versus Rest ROC (Receiver Operating Characteristic Curve) and AUC measures.

## 3 Result

### 3.1 Demographic Characteristics of Participants

The demographic characteristics, diagnostic assessments, and developmental evaluation outcomes of all participants are summarized in Table 1. Regarding social‐behavioral measures, children with ASD demonstrated significantly higher scores than the FXS group on both the CSS (*p* < 0.001) and SA (*p* < 0.001) domains of ADOS‐2, while no between‐group difference emerged in RRB scores (*p* = 0.53). The ASD group showed significantly higher scores than the FXS group across multiple domains of the Griffiths Developmental Scales, including GM (*p* < 0.001), FM (*p* = 0.021), and language (*p* = 0.046).

**TABLE 1:** Demographic characteristics of the participants.

	TD	ASD	FXS	*p*
Age (years)	5.2 ± 2.9	3.4 ± 1.4	5.2 ± 2.5	< 0.001^a^
Sex (male/female)	36/16	63/28	44/3	0.004^b^
ADOS‐2				
CSS‐total	/	6.85 ± 1.68	5.33 ± 2.53	< 0.001^c^
SA	/	14.69 ± 3.95	10.37 ± 5.71	< 0.001^c^
RRB	/	2.83 ± 1.60	3.04 ± 2.25	0.53^c^
GDS‐C				
Gross Motor	/	73.95 ± 14.35	61.64 ± 15.42	0.001^a^
Personal‐Social	/	55.79 ± 15.16	49.85 ± 20.62	0.086^a^
Language	/	44.84 ± 18.13	41.45 ± 26.11	0.423^a^
Fine Motor	/	64.07 ± 15.15	55.82 ± 21.92	0.021^a^
Performance	/	66.42 ± 18.04	58.76 ± 20.02	0.046^a^

Abbreviations: ASD, autism spectrum disorder; CSS, comparison score; FXS, fragile X syndrome; GDS‐C, Griffiths developmental scales‐Chinese; RRB, restricted repetitive behaviors; SA, social affect; TD, typical development.

^a^ *t*‐test.

^b^ Chi‐square test.

^c^ Mann–Whitney *U* test.

### 3.2 Pattern of Dynamic Functional Connectivity

**FIGURE 1:**
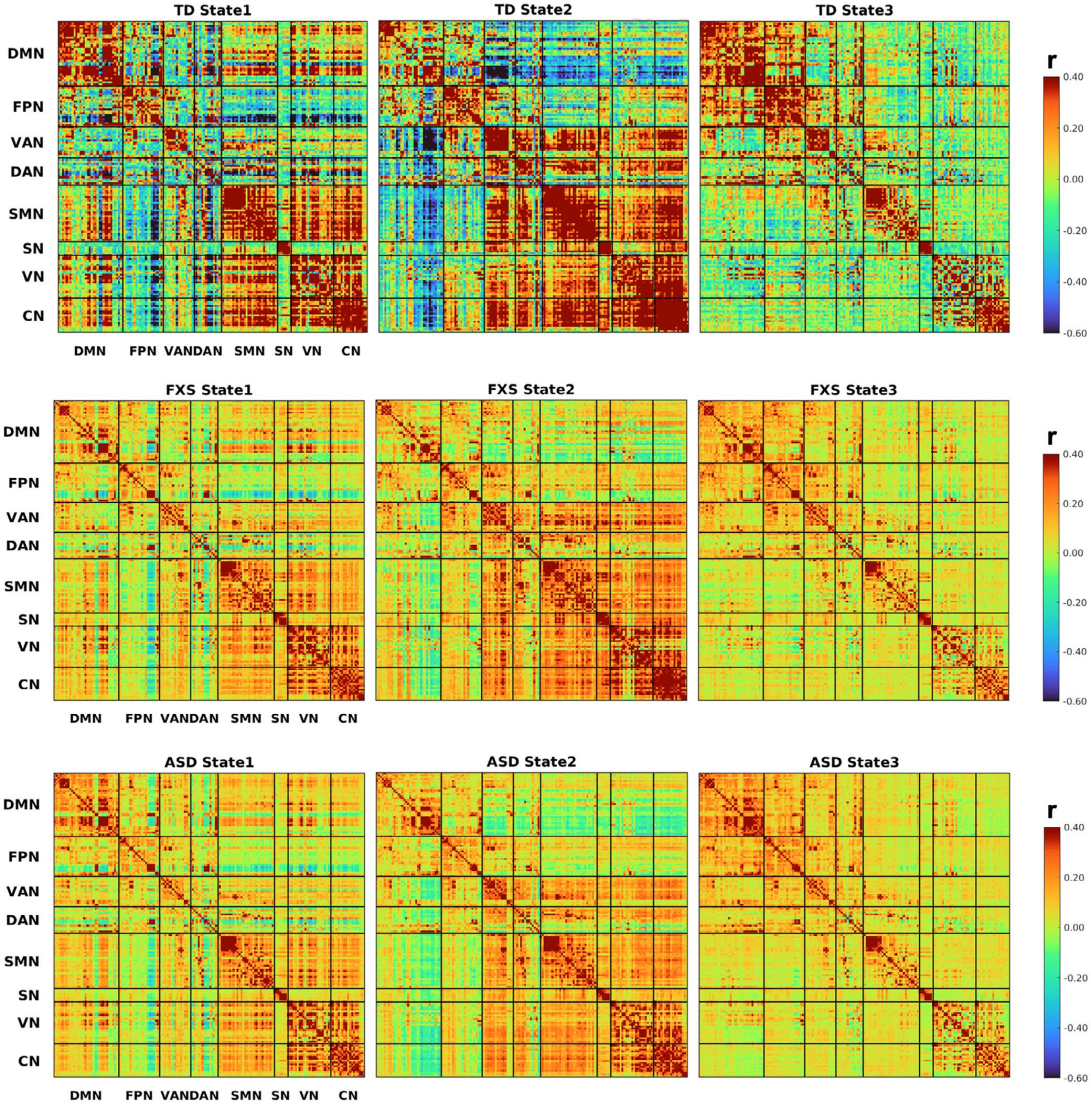
Dynamic functional connectivity states of the TD, ASD, and FXS groups. CN, cerebellum network; DAN, dorsal attention network; DMN, default mode network; FPN, frontoparietal network; SMN, sensorimotor network; SN, subcortical network; VAN, ventral attention network; VN, visual network. The color bar adjacent to the heatmap indicates the functional connectivity strength.

Using the k‐means clustering method, we identified three highly structured dFC states that recurred throughout individual scans and across all participants (Figure 1). To classify each of the three states into a corresponding categorical pattern, we obtained the mean FC matrix of each TD child across windows. Later, the global mean FC was computed as the average of all mean FC matrices across the TD children. Our thresholds were then determined by the average and standard deviation (0.453 ± 0.268) of the global mean FC matrix [50]. Hence, each of the three categorical patterns (i.e., relatively sparse connectivity, intermediate connectivity, and relatively strong connectivity) was identified by the higher proportion governed by these thresholds (as shown in Table 2). Consequently, our three states were observed to fall into the following categories: State 1, a relatively strong connectivity pattern; State 2, an intermediate connectivity pattern; and State 3, a relatively sparse connectivity pattern.

**TABLE 2:** Proportions of the three types of connectivity in all states.

State	Relatively sparse connectivity (|correlation| < 0.2)	Intermediate connectivity (0.2 < |correlation| < 0.6)	Relatively strong connectivity (|correlation| > 0.6)
State 1	0.472	0.425	0.101
State 2	0.437	0.480	0.083
State 3	0.668	0.281	0.051

### 3.3 Temporal Properties of Dynamic Functional Connectivity

**FIGURE 2:**
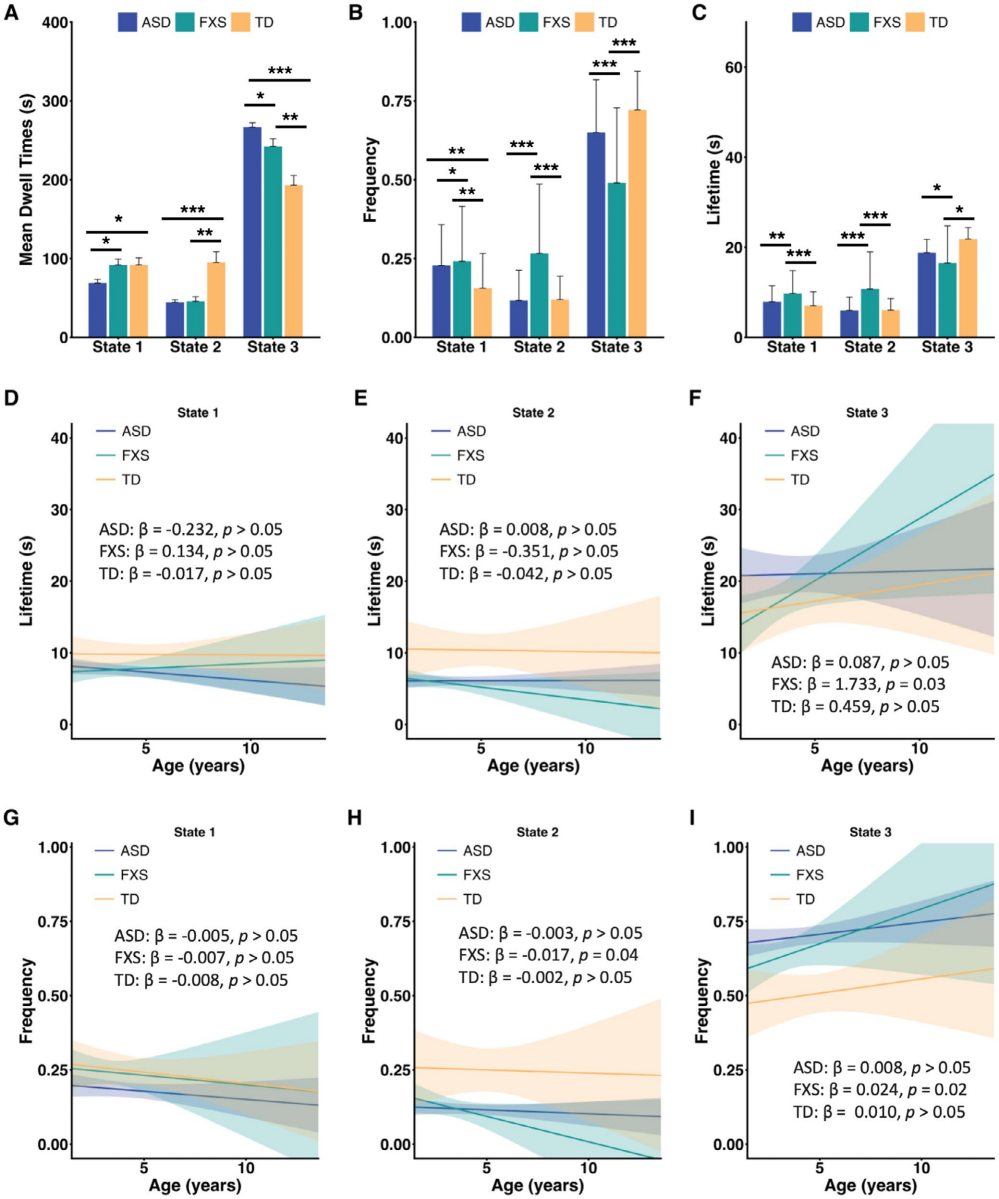
The temporal properties of dynamic functional connectivity in the TD, ASD, and FXS groups. (A) Dwell time (state duration). (B) Transition frequency (number of shifts between states). (C) Lifetimes (persistence of state sequences). (D) The relationship between lifetimes and age in children from different groups in State 1. (E) The relationship between lifetimes and age in children from different groups in State 2. (F) The relationship between lifetimes and age in children from different groups in State 3. The shaded area represents the 95% confidence interval. (G) The relationship between transition frequency and age in children from different groups in State 1. (H) The relationship between transition frequency and age in children from different groups in State 2. (I) The relationship between transition frequency and age in children from different groups in State 3. The shaded area represents the 95% confidence interval. *p < 0.05, **p < 0.01, ***p < 0.001 (FDR correction).

Dwell time across brain states showed distinct patterns among groups (Figure 2A). In State 1, the dwell time of the ASD group was significantly lower than that of the TD (*p* < 0.05, FDR correction) and FXS groups (*p* < 0.05, FDR correction). In State 2, the dwell time of the TD group was significantly higher than that of the FXS (*p* < 0.01, FDR correction) and ASD groups (*p* < 0.001, FDR correction). Both the dwell time of the ASD and FXS groups in State 3 are higher than that of the TD group (ASD: *p* < 0.001, FXS: *p* < 0.01, FDR correction), and the ASD group also showed significantly longer dwell time than the FXS group (*p* < 0.05, FDR correction).

Meanwhile, the transition frequencies between brain states reveal different patterns among groups (Figure 2B). The transition frequency from State 1 to other states in the TD group was significantly lower than in the ASD group (*p* < 0.01, FDR correction) and the FXS group (*p* < 0.01, FDR correction), but there was no significant difference between the ASD and FXS groups. Both the ASD and TD groups showed significantly lower transition frequencies in State 2 compared to the FXS group (ASD: *p* < 0.001, TD: *p* < 0.001, FDR correction), but significantly higher transition frequencies in State 3 compared to the FXS group (ASD: *p* < 0.001, TD: *p* < 0.001, FDR correction).

Figure 2C shows the differences in lifetimes across states for each group. In State 1 and State 2, the lifetimes of the ASD and TD groups were shorter compared to the FXS group (State 1: ASD: *p* < 0.05, TD: *p* < 0.01; State 2: ASD: *p* < 0.001, TD: *p* < 0.001, FDR correction). In State 3, the lifetimes of the FXS group were significantly shorter than those of the TD group (*p* < 0.05, FDR correction).

### 3.4 Variations in Temporal Properties Across Child Developmental Stages

The regression analysis showed that with increasing age, the lifetime of FXS children exhibited a consistent upward trend in State 3 (*β* = 1.733, *p* = 0.03; Figure 2F). However, the FXS group did not show a similar trend in State 1 (*β* = 0.134, *p* > 0.05; Figure 2D) and State 2 (*β* = −0.351, *p* > 0.05; Figure 2E), whereas the ASD and TD groups did not show a similar trend in all States. The transition frequency of FXS children showed a continuous decrease in State 2 (*β* = −0.017, *p* = 0.04; Figure 2H) and an increase in State 3 (*β* = 0.024, *p* = 0.02; Figure 2I) as age increased. But no significant trend in the FXS group was found in State 1 (*β* = −0.007, *p* > 0.05; Figure 2G). Notably, such trends in transition frequency were absent in both the ASD and TD groups. For dwell time, such trends were not observed in children across groups.

### 3.5 Topological Properties of Dynamic Functional Connectivity Across States

**FIGURE 3:**
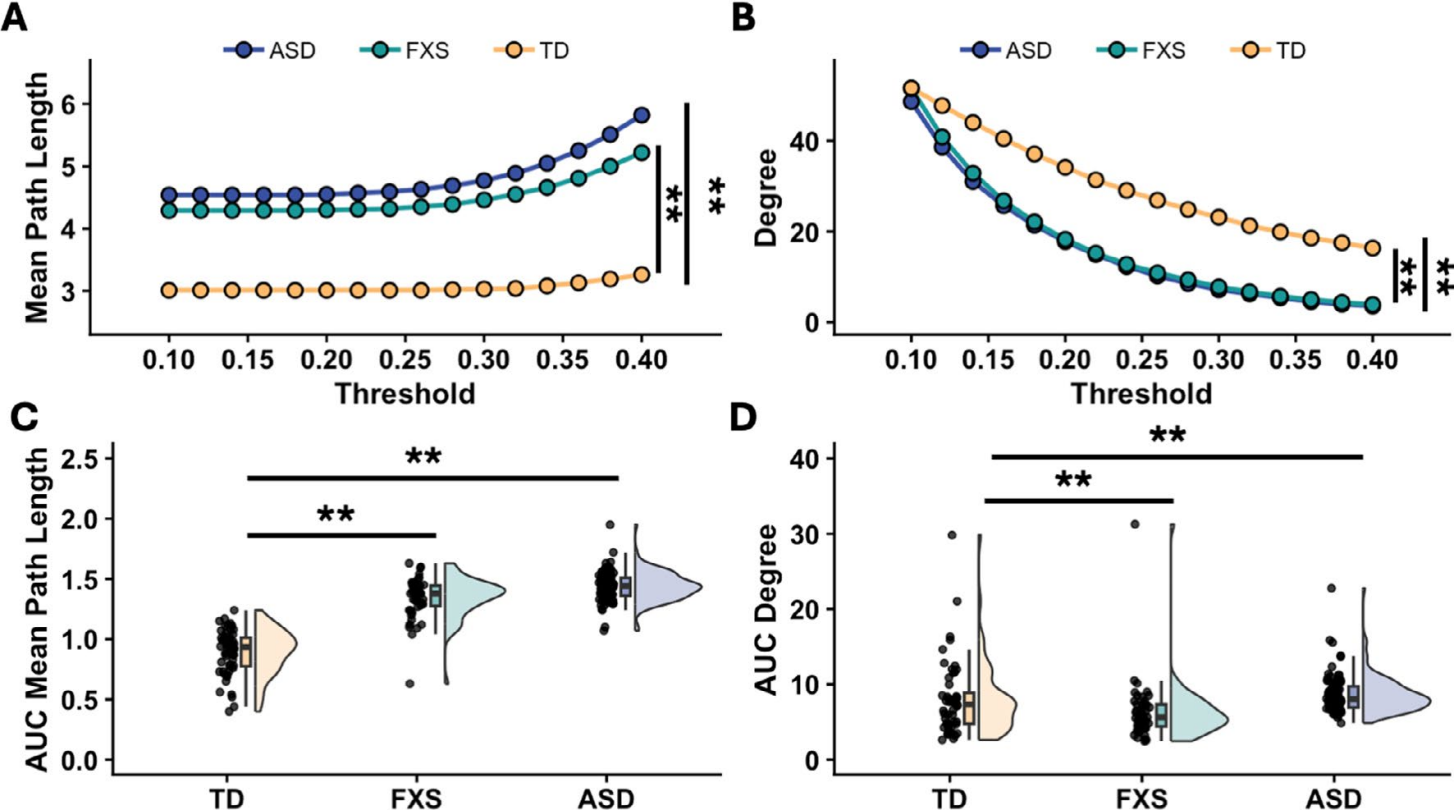
Differences in AUC of topological properties across groups in State 3. (A) The AUC of path length of different groups in State 3. (B) The AUC of degree of different groups in State 3. (C) The path length AUC distribution of children in different groups. (D) The degree AUC distribution of children in different groups. **p < 0.01.

Topological analysis revealed that differences in topological properties among different groups were concentrated in State 3. The AUC for degree and path length was calculated across a sparsity threshold range (0.1–0.4, step = 0.02). For path length (Figure 3A), the ASD and FXS groups showed markedly diminished AUC in comparison to the TD group (ASD: *p* < 0.01, FXS: *p* < 0.01), but there was no significant difference in AUC between the ASD and FXS groups. For degree (Figure 3B), a significantly lower AUC was observed in the ASD and FXS groups relative to the TD group (ASD: *p* < 0.01, FXS: *p* < 0.01), with no significant difference between the ASD and FXS groups. The path length AUC distribution and the degree AUC distribution for children across different groups are shown in Figure 3C,D, respectively.

### 3.6 Normative Modeling of Intrinsic Networks

**FIGURE 4:**
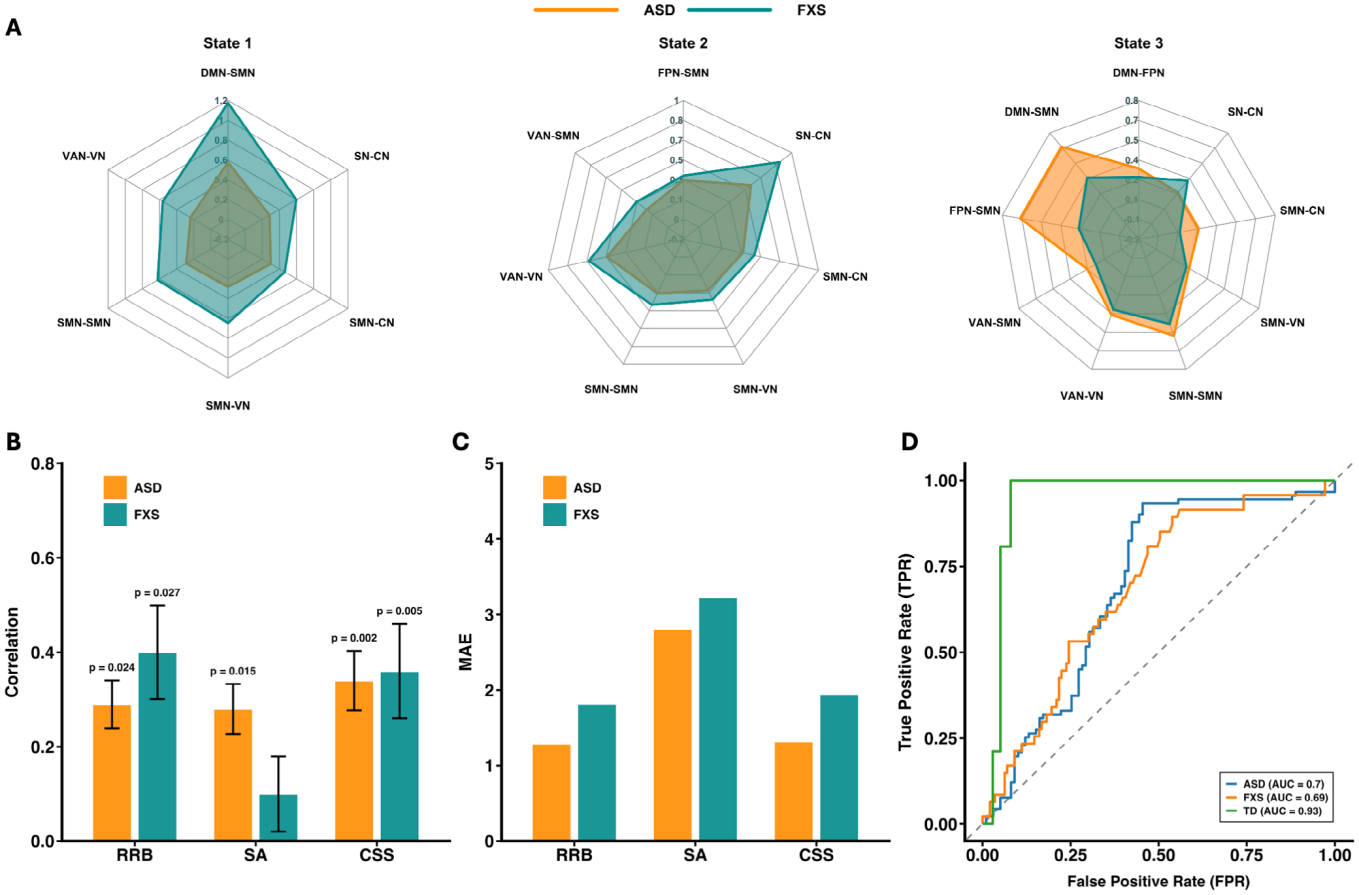
(A) The deviation of FC patterns using the normative modeling, with values corresponding to the Cohen's *d* of network connections. (B) The correlation coefficient between the predicted scores and true scores of ADOS‐2. (C) The prediction accuracies and MAE of ADOS‐2 based on deviations of FC patterns. CSS, comparison score; RRB, restricted repetitive behaviors; SA, social affect. (D) ROC curve and AUC for classification of ASD, FXS, and TD groups.

The normative modeling identified FC patterns that exhibited significant differences between the ASD and FXS groups relative to the TD group, with corresponding Cohen's *d* values shown in Figure 4A. In State 1, both ASD and FXS groups demonstrated similar hyperconnectivity across multiple networks, including DMN‐SMN (ASD: *p* < 0.001; FXS: *p* < 0.001), SN‐CN (ASD: *p* = 0.008; FXS: *p* < 0.001), SMN‐CN (ASD: *p* = 0.005; FXS: *p* = 0.003), SMN‐VN (ASD: *p* = 0.009; FXS: *p* < 0.001), SMN‐SMN (ASD: *p* = 0.007; FXS: *p* < 0.001), and VAN‐VN (ASD: *p* = 0.025; FXS: *p* < 0.001). The DMN‐SMN connectivity exhibited the most pronounced deviation in both groups (ASD: Cohen's *d* = 0.58; FXS: Cohen's *d* = 1.17).

Similarly, in State 2, both groups displayed overlapping hyperconnectivity patterns predominantly involving the SMN. Significant differences were observed in FPN‐SMN (ASD: *p* = 0.004; FXS: *p* = 0.0221), VAN‐SMN (ASD: *p* = 0.047; FXS: *p* = 0.033), VAN‐VN (ASD: *p* < 0.001; FXS: *p* < 0.001), SMN‐SMN (ASD: *p* = 0.0032; FXS: *p* = 0.0049), SMN‐VN (ASD: *p* = 0.007; FXS: *p* = 0.013), SMN‐CN (ASD: *p* = 0.003; FXS: *p* = 0.005), and SN‐CN (ASD: *p* < 0.001; FXS: *p* < 0.001). Cohen's *d* values were generally higher in the FXS group.

In State 3, the ASD and FXS groups exhibited partially overlapping but distinct connectivity profiles. Shared hyperconnectivity was observed in DMN‐SMN (ASD: *p* < 0.001; FXS: *p* = 0.012), VAN‐VN (ASD: *p* < 0.001; FXS: *p* = 0.023), SMN‐SMN (ASD: *p* < 0.001; FXS: *p* = 0.003), and SN‐CN (ASD: *p* = 0.027; FXS: *p* = 0.019). The ASD group showed larger effect sizes for the first three connections: DMN‐SMN (Cohen's *d* = 0.67), VAN‐VN (Cohen's *d* = 0.383), and SMN‐SMN (Cohen's *d* = 0.539). Additionally, the ASD group displayed unique SMN‐centric hyperconnectivity in DMN‐FPN (*p* = 0.004), FPN‐SMN (*p* < 0.001), VAN‐SMN (*p* = 0.0342), SMN‐VN (*p* = 0.042), and SMN‐CN (*p* = 0.027), resembling patterns observed in State 2.

### 3.7 Prediction of ASD Symptoms Based on Deviations of Intrinsic Network FC

The association between each ADOS‐2 domain and the deviation of intrinsic network FC is shown in Figure 4B. For the ASD children, we observed significant correlations with CSS (*r* = 0.34 ± 0.063, *p* = 0.002), RRB (*r* = 0.29 ± 0.051, *p* = 0.024), and SA scores (*r* = 0.28 ± 0.053, *p* = 0.015). For the FXS children, significant correlations were found for the CSS (*r* = 0.36 ± 0.1, *p* = 0.005) and RRB scores (*r* = 0.40 ± 0.099, *p* = 0.027). The MAE of each domain of ADOS‐2 through the deviation of intrinsic network FC is shown in Figure 4C. For the RRB domain, the model for children with ASD achieved an MAE of 1.29, whereas the model for children with FXS achieved an MAE of 1.82. In the SA domain, the ASD model achieved an MAE of 2.81, compared to 3.23 for the FXS model. Regarding CSS, the MAE was 1.32 for the ASD model and 1.94 for the FXS model.

### 3.8 Classification Based on Deviations of Intrinsic Network FC

Using the *Z* scores of deviations from all participants, we trained an elastic‐net classification model with five‐fold cross‐validation. The resulting three‐class model achieved an accuracy of 67.4% ± 3%, with class‐specific AUC values of 0.70 for the ASD group, 0.69 for the FXS group, and 0.93 for the TD group. Figure 4D illustrates the ROC and AUC of the classification.

## 4 Discussion

In this study, we used dynamic FC analysis and normative modeling to investigate the dynamic brain network characteristics of ASD and FXS, and to examine whether individualized dFC could predict the core autistic symptoms of children with ASD and FXS. We identified distinct disturbances in the temporal properties and topological network properties in both ASD and FXS cohorts, with some shared patterns between these two phenotypically overlapping groups. Our normative modeling analysis further quantitatively delineated patterns of shared and syndrome‐specific neurophysiological heterogeneity in these two cohorts. These heterogeneous and individualized network metrics also showed significant correlations with their clinical measurements. Overall, the dFC abnormalities revealed by our study advance the understanding of neural mechanisms underlying similar cognitive‐behavioral impairments in ASD and FXS, further facilitating the development of individualized treatment for these neurodevelopmental disorders.

Our analysis identified three distinct dFC states, including a relatively strong FC pattern (State 1), an intermediate FC pattern (State 2), and a relatively sparse FC pattern (State 3). Compared to TD children, ASD children showed reduced dwell time in State 1 and State 2 and longer dwell time in State 3. This is consistent with prior studies showing that traits of delayed reactions and thought processes in ASD children [16, 51]. We hypothesized that the observed reduced dwell time and increased transition frequency in State 1 may be attributed to the impaired network integration in ASD children, a potential cause of their cognitive rigidity [3]. Although FXS children shared some temporal metric abnormalities with ASD, they exhibited unique flexibility in States 2 and 3, as evidenced by higher frequency and longer lifetimes in State 2 and lower frequency and shorter lifetimes in State 3. This distinction may reflect differences in FMR1‐related synaptic dysfunction in FXS compared to the transitional state dysfunction in ASD.

We identified a distinct dynamic developmental pattern in the FXS group. Although FXS children displayed aberrant temporal characteristics in State 2 and State 3, they exhibited age‐related increases in lifetimes and frequency in State 3, together with lower frequency in State 2. These age‐dependent alterations suggest that the developmental trajectory of FXS children is, to some extent, more similar to those observed in typically developing children. Such patterns may indicate partially preserved or compensatory maturation of large‐scale network dynamics in FXS [33, 43]. In contrast, the absence of similar trends in children with ASD may indicate disrupted or delayed maturation of brain dynamics [1, 2].

The topological analysis showed that ASD and FXS children exhibited significantly lower degree AUC and higher path length AUC, which is consistent with previous studies [20, 21]. The reduced degree indicates suppressed local FC density and weakened interregional integration, while the enhanced path length implies a disrupted balance between network segregation and integration [52, 53]. This shift in topological property, according to the small‐world network theory, suggests that the brain development of ASD or FXS children deviates from the optimized small‐world architecture toward more randomized configurations [52]. It is believed that strategic pruning of redundant neuronal connections and strengthening of core neural pathways progressively refine network efficiency in typical brain development [54]. And our findings of the random‐like topology in FXS and ASD groups may therefore stem from the dysregulated developmental pruning processes.

The normative model showed that ASD and FXS children exhibited enhanced FC between SMN‐SMN, VAN‐VN, and SN‐CN across states. The Enhanced Perceptual Functioning (EPF) theory supports that sensory processing in ASD is characterized by overly detailed encoding alongside a deficit in contextual integration [55]. Considering that the VAN plays a major role in primary visual perception [56], the heightened connectivity between VAN and VN may reflect over‐driven bottom‐up mechanisms [57]. Given the high comorbidity of attention‐deficit/hyperactivity disorder (ADHD) symptoms and emotional dysregulation in both ASD and FXS, it is also possible that the dysconnectivity observed in the VAN and DMN in different states may partly reflect emotional dysregulation. Emerging evidence has shown that emotional dysregulation in ADHD is associated with altered connectivity between the right ventrolateral prefrontal cortex (VLPFC) and the left amygdala. Therefore, the identified VAN‐ and DMN‐related alterations may be also related to the altered VLPFC and left amygdala in children with comorbid ADHD [58, 59]. Previous studies have identified volume alterations in SN and CN as among the most prevalent neuroanatomical abnormalities in ASD and FXS [60, 61]. The observed SN‐CN hyperconnectivity may be associated with these volumetric abnormalities.

Using elastic net regression and classification, the individualized deviations in dFC demonstrated strong predictive power for clinical symptoms of ASD and FXS (Figure 4). Specifically, the methodological framework proposed in this study can correlate the dFC characteristics to the behavioral manifestations in children with ASD and FXS, suggesting that individualized dFC metrics with TD as baseline are significantly associated with disease‐specific clinical characteristics. From a translational perspective, the derived dFC patterns could serve as a preliminary neurophysiological basis for future efforts to monitor treatment efficacy in clinical trials and to inform personalized therapeutic strategies, addressing the inherent limitations in clinical assessments due to the overlap of typical behaviors in ASD and FXS. However, these applications remain speculative at this stage and will require validation in independent cohorts.

Some limitations should be acknowledged in the current study. First, although we included age as a covariate in the statistical analysis, the age difference between the ASD group and other groups may still bias the difference in dynamic functional connectivity. Second, FXS is a typical X‐linked disorder caused by skewed X‐chromosome mutation, the imbalanced gender ratio among the three groups may bring interpretative constraints on the findings in the current study. In addition, ASD generally presents high heterogeneity, as evidenced by a range of subtypes and common comorbidities in the related cohort. Future studies should balance age and sex distributions, expand sample sizes, and incorporate more demographic measurements into the normative models. It would also be valuable to develop subtype‐specific models for different ASD and FXS phenotypes, further advancing the development of individualized treatment for the affected individuals.

## Funding

This work was supported by the National Natural Science Foundation of China, 82301743; the Science and Technology Development Fund of the Macao SAR, 0010/2023/ITP1, 0016/2024/RIB1; Natural Science Foundation of Guangdong Province, 2025A1515010539; the University of Macau, SRG2023‐00015‐ICI, MYRG‐GRG2024‐00296‐ICI, MYRG‐CRG2024‐00022‐ICI; the Natural Science Foundation of Anhui Province, 2308085MH255; the academic leaders development program, EKXDPY202306; Med+X cross‐disciplinary team project of the Children's Hospital of Fudan University.

## Ethics Statement

The study was conducted in accordance with the principles of the Declaration of Helsinki and received approval from the Ethics Committee of Children's Hospital of Fudan University (approval number: 2021‐312).

## Consent

Written informed consent was obtained from all participants or their legal guardians after they were thoroughly informed of the study procedures and objectives.

## Conflicts of Interest

The authors declare no conflicts of interest.

## Supporting information


**Table S1:** Regions and MNI coordinates of 160 ROIs.

## Data Availability

The data that support the findings of this study are available on request from the corresponding author. The data are not publicly available due to privacy or ethical restrictions.
