# Supplementary material for: Unraveling the Individualized Shared and Distinct Dynamic Functional Connectivity in Idiopathic Autism Spectrum Disorder and Fragile X Syndrome
Source: CNS Neurosci Ther. 2026 Jul 24;32(7):e71046. doi: 10.1002/cns.71046 (PMC13398155; doi:10.1002/cns.71046)
Supplement: Supplementary file 1 — Table S1: Regions and MNI coordinates of 160 ROIs. [file CNS-32-e71046-s001.docx]

**SUPPLEMENTARY INFORMATION**

**Individual evaluation of ASD and FXS children atypical dynamic functional brain network based on normative models**

Pan *et al*.

**Identified 160 functional ROIs**

In the current study, we identified eight intrinsic functional networks using a well-established functional parcellation derived from a sample of 1000 healthy individuals. These networks comprising 160 ROIs, including sensorimotor network (SMN), ventral attention network (VAN), visual network (VN), dorsal attention network (DAN), default mode network (DMN), frontoparietal network (FPN), subcortical network (SN) and cerebellum network (CN). Table S1 shows the regions and Montreal Neurological Institute (MNI) coordinates of 160 ROIs.

Table S1 Regions and MNI coordinates of 160 ROIs

| **Identified 160 functional ROIs** | | | | |
| --- | --- | --- | --- | --- |
| **X** | **Y** | **Z** | **ROI Label** | **Network** |
| 6 | 64 | 3 | vmPFC' | DMN' |
| 29 | 57 | 18 | aPFC' | FPN' |
| -29 | 57 | 10 | aPFC' | FPN' |
| 0 | 51 | 32 | mPFC' | DMN' |
| -25 | 51 | 27 | aPFC' | DMN' |
| 9 | 51 | 16 | vmPFC' | DMN' |
| -6 | 50 | -1 | vmPFC' | DMN' |
| 27 | 49 | 26 | aPFC' | VAN' |
| 42 | 48 | -3 | vent aPFC' | FPN' |
| -43 | 47 | 2 | vent aPFC' | FPN' |
| -11 | 45 | 17 | vmPFC' | DMN' |
| 39 | 42 | 16 | vlPFC' | FPN' |
| 8 | 42 | -5 | vmPFC' | DMN' |
| 9 | 39 | 20 | ACC' | DMN' |
| 46 | 39 | -15 | vlPFC' | DMN' |
| 40 | 36 | 29 | dlPFC' | FPN' |
| 23 | 33 | 47 | sup frontal' | DMN' |
| 34 | 32 | 7 | vPFC' | VAN' |
| -2 | 30 | 27 | ACC' | FPN' |
| -16 | 29 | 54 | sup frontal' | DMN' |
| -1 | 28 | 40 | ACC' | FPN' |
| 46 | 28 | 31 | dlPFC' | FPN' |
| -52 | 28 | 17 | vPFC' | FPN' |
| -44 | 27 | 33 | dlPFC' | FPN' |
| 51 | 23 | 8 | vFC' | DMN' |
| 38 | 21 | -1 | ant insula' | VAN' |
| 9 | 20 | 34 | dACC' | VAN' |
| -36 | 18 | 2 | ant insula' | VAN' |
| 40 | 17 | 40 | dFC' | FPN' |
| -6 | 17 | 34 | basal ganglia' | VAN' |
| 0 | 15 | 45 | mFC' | VAN' |
| 58 | 11 | 14 | frontal' | VAN' |
| -46 | 10 | 14 | vFC' | FPN' |
| 44 | 8 | 34 | dFC' | DAN' |
| 60 | 8 | 34 | dFC' | SMN' |
| -42 | 7 | 36 | dFC' | FPN' |
| -55 | 7 | 23 | vFC' | DAN' |
| -20 | 6 | 7 | basal ganglia' | SN' |
| 14 | 6 | 7 | basal ganglia' | SN' |
| -48 | 6 | 1 | vFC' | VAN' |
| 10 | 5 | 51 | pre-SMA' | VAN' |
| 43 | 1 | 12 | vFC' | VAN' |
| 0 | -1 | 52 | SMA' | SMN' |
| 37 | -2 | -3 | mid insula' | VAN' |
| 53 | -3 | 32 | frontal' | SMN' |
| 58 | -3 | 17 | precentral gyrus' | SMN' |
| -12 | -3 | 13 | thalamus' | SN' |
| -42 | -3 | 11 | mid insula' | VAN' |
| -44 | -6 | 49 | precentral gyrus' | DAN' |
| -26 | -8 | 54 | parietal' | DAN' |
| 46 | -8 | 24 | precentral gyrus' | SMN' |
| -54 | -9 | 23 | precentral gyrus' | SMN' |
| 44 | -11 | 38 | precentral gyrus' | SMN' |
| -47 | -12 | 36 | parietal' | SMN' |
| 33 | -12 | 16 | mid insula' | SMN' |
| -36 | -12 | 15 | mid insula' | SMN' |
| -12 | -12 | 6 | thalamus' | SN' |
| 11 | -12 | 6 | thalamus' | SN' |
| 32 | -12 | 2 | mid insula' | SMN' |
| 59 | -13 | 8 | temporal' | SMN' |
| -30 | -14 | 1 | mid insula' | SMN' |
| -38 | -15 | 59 | parietal' | SMN' |
| 52 | -15 | -13 | inf temporal' | DMN' |
| -47 | -18 | 50 | parietal' | SMN' |
| 46 | -20 | 45 | parietal' | SMN' |
| -55 | -22 | 38 | parietal' | DAN' |
| -54 | -22 | 22 | precentral gyrus' | SMN' |
| -54 | -22 | 9 | temporal' | SMN' |
| 41 | -23 | 55 | parietal' | SMN' |
| 42 | -24 | 17 | post insula' | SMN' |
| 11 | -24 | 2 | basal ganglia' | SN' |
| -59 | -25 | -15 | inf temporal' | DMN' |
| 1 | -26 | 31 | post cingulate' | DMN' |
| 18 | -27 | 62 | parietal' | SMN' |
| -38 | -27 | 60 | parietal' | SMN' |
| -30 | -28 | 9 | post insula' | SMN' |
| -24 | -30 | 64 | parietal' | SMN' |
| 51 | -30 | 5 | temporal' | SMN' |
| -41 | -31 | 48 | post parietal' | DAN' |
| -4 | -31 | -4 | post cingulate' | SN' |
| 54 | -31 | -18 | fusiform' | FPN' |
| -41 | -37 | 16 | temporal' | SMN' |
| -53 | -37 | 13 | temporal' | SMN' |
| 28 | -37 | -15 | fusiform' | VN' |
| -3 | -38 | 45 | precuneus' | DMN' |
| 34 | -39 | 65 | sup parietal' | SMN' |
| 8 | -40 | 50 | precuneus' | DAN' |
| -41 | -40 | 42 | IPL' | DAN' |
| 58 | -41 | 20 | parietal' | VAN' |
| -8 | -41 | 3 | post cingulate' | DMN' |
| -61 | -41 | -2 | inf temporal' | DMN' |
| -28 | -42 | -11 | occipital' | VN' |
| -5 | -43 | 25 | post cingulate' | DMN' |
| 9 | -43 | 25 | precuneus' | DMN' |
| 43 | -43 | 8 | temporal' | VAN' |
| 54 | -44 | 43 | IPL' | FPN' |
| -55 | -44 | 30 | parietal' | VAN' |
| -28 | -44 | -25 | lat cerebellum' | CN' |
| -35 | -46 | 48 | post parietal' | DAN' |
| 42 | -46 | 21 | sup temporal' | DMN' |
| -48 | -47 | 49 | IPL' | FPN' |
| -41 | -47 | 29 | angular gyrus' | DMN' |
| -59 | -47 | 11 | temporal' | SMN' |
| -53 | -50 | 39 | IPL' | DMN' |
| 5 | -50 | 33 | precuneus' | DMN' |
| -18 | -50 | 1 | occipital' | VN' |
| 44 | -52 | 47 | IPL' | FPN' |
| -5 | -52 | 17 | post cingulate' | DMN' |
| -24 | -54 | -21 | lat cerebellum' | CN' |
| -37 | -54 | -37 | inf cerebellum' | CN' |
| 10 | -55 | 17 | post cingulate' | DMN' |
| -6 | -56 | 29 | precuneus' | DMN' |
| -34 | -57 | -24 | lat cerebellum' | CN' |
| -32 | -58 | 46 | IPS' | FPN' |
| -11 | -58 | 17 | post cingulate' | DMN' |
| 32 | -59 | 41 | IPS' | DAN' |
| 51 | -59 | 34 | angular gyrus' | DMN' |
| -34 | -60 | -5 | occipital' | VN' |
| 36 | -60 | -8 | occipital' | VN' |
| -6 | -60 | -15 | med cerebellum' | CN' |
| -25 | -60 | -34 | inf cerebellum' | CN' |
| 32 | -61 | -31 | inf cerebellum' | CN' |
| 46 | -62 | 5 | temporal' | VN' |
| -48 | -63 | 35 | angular gyrus' | DMN' |
| -52 | -63 | 15 | TPJ' | DAN' |
| -44 | -63 | -7 | occipital' | DAN' |
| -16 | -64 | -21 | med cerebellum' | CN' |
| 21 | -64 | -22 | lat cerebellum' | CN' |
| 19 | -66 | -1 | occipital' | VN' |
| 1 | -66 | -24 | med cerebellum' | CN' |
| -34 | -67 | -29 | inf cerebellum' | CN' |
| 11 | -68 | 42 | precuneus' | DMN' |
| 17 | -68 | 20 | occipital' | VN' |
| -36 | -69 | 40 | IPS' | DMN' |
| 39 | -71 | 13 | occipital' | VN' |
| -9 | -72 | 41 | occipital' | FPN' |
| 45 | -72 | 29 | occipital' | DMN' |
| -11 | -72 | -14 | med cerebellum' | CN' |
| 29 | -73 | 29 | occipital' | VN' |
| 33 | -73 | -30 | inf cerebellum' | CN' |
| -2 | -75 | 32 | occipital' | FPN' |
| -29 | -75 | 28 | occipital' | DAN' |
| 5 | -75 | -11 | med cerebellum' | CN' |
| 14 | -75 | -21 | med cerebellum' | CN' |
| -16 | -76 | 33 | occipital' | VN' |
| -42 | -76 | 26 | occipital' | DAN' |
| 9 | -76 | 14 | occipital' | VN' |
| 15 | -77 | 32 | occipital' | VN' |
| 20 | -78 | -2 | occipital' | VN' |
| -21 | -79 | -33 | inf cerebellum' | CN' |
| -6 | -79 | -33 | inf cerebellum' | CN' |
| -5 | -80 | 9 | post occipital' | VN' |
| 29 | -81 | 14 | post occipital' | VN' |
| 33 | -81 | -2 | post occipital' | VN' |
| 18 | -81 | -33 | inf cerebellum' | CN' |
| -37 | -83 | -2 | post occipital' | VN' |
| -29 | -88 | 8 | post occipital' | VN' |
| 13 | -91 | 2 | post occipital' | VN' |
| 27 | -91 | 2 | post occipital' | VN' |
| -4 | -94 | 12 | post occipital' | VN' |

**Topological Metrics**

The shortest weighted path length between nodes i and j. d_ij_^w^ was defined as:

$$\boldsymbol{d}_{\boldsymbol{i,j}}^{\boldsymbol{w}}\boldsymbol{=}\sum_{\boldsymbol{a}_{\boldsymbol{u,v}}\boldsymbol{\in}\boldsymbol{g}_{\boldsymbol{i+j}}^{\boldsymbol{w}}} \boldsymbol{f}\left( \boldsymbol{w}_{\boldsymbol{uv}} \right)$$

Where a_uv_ was the connection status; a_uv_ = 1 as a link (*i*, *j*) existed (i.e., node *i* and *j* are neighbors); otherwise a_uv_ = 0. The term w_uv_ stood for the connection weights between nodes *u* and *v*. The function *f* maps a weight value to its corresponding length, typically as an inverse mapping. g_i↔︎j_^w^ denoted the shortest weighted path between node *i* and *j*.

We calculated degree of nodal centrality for each of the 160 nodes. Degree was the sum of links’ weights connected to a node. which was computed as:

$$k_{i}^{w}=\sum_{j\in N} w_{ij}$$

Where *k_i_^w^*​ is the **weighted degree**of node i*i*, defined as the **sum of the weights** of all edges connected to node *i*. *w_ij​_* is the **connection weight** between nodes *ii* and *jj*
